# Poster Session II A331 AGE AND SEX INFLUENCE ESOPHAGEAL SYMPTOMS IN REFLUX-NEGATIVE PATIENTS WITH NORMAL ESOPHAGEAL MOTILITY

**DOI:** 10.1093/jcag/gwaf042.331

**Published:** 2026-02-13

**Authors:** R Kharfan, K Pokraka, D Y Li, M Buresi, C N Andrews, Y Nasser, M Gupta, M Woo

**Affiliations:** Internal Medicine, University of Alberta, Edmonton, AB, Canada; University of Manitoba Max Rady College of Medicine, Winnipeg, MB, Canada; Medicine, University of Calgary, Calgary, AB, Canada; Medicine, University of Calgary, Calgary, AB, Canada; Medicine, University of Calgary, Calgary, AB, Canada; Medicine, University of Calgary Cumming School of Medicine, Calgary, AB, Canada; Medicine, University of Calgary, Calgary, AB, Canada; Medicine, University of Calgary, Calgary, AB, Canada

## Abstract

**Background:**

Functional esophageal symptoms such as heartburn and dysphagia are common, often occurring in the absence of demonstrable reflux or major motility disorders. Pathophysiology is multifactorial, involving visceral hypersensitivity, altered central processing, and impaired esophageal perception. The relationship between demographic factors and symptom burden in patients with normal motility and no reflux remains unclear. The Esophageal Symptom Questionnaire (ESQ-30) is a validated tool measuring frequency and severity of dysphagia (ESQ-D), reflux (ESQ-R), and globus (ESQ-G).

**Aims:**

To determine the effect of age and sex on esophageal symptoms in reflux-negative patients with normal esophageal motility.

**Methods:**

We retrospectively analyzed consecutive high-resolution manometry (HRM) studies between July 2022–October 2024. Inclusion required normal esophageal motility (no esophageal motility disorder by Chicago Classification v4.0) and complete ESQ-30 data. Patients with evidence of GERD by Lyon 2.0 criteria (LA grade B-D esophagitis/Barrett’s esophagus, off-therapy AET >6%, on-therapy AET >4%) were excluded. ESQ subscales were summarized as median (IQR). Group comparisons used Mann-Whitney U tests. Correlations between ESQ subscales, manometric variables (IRP, DCI, % failed, % weak, % incomplete bolus clearance), and age were assessed with Spearman’s bivariate correlations.

**Results:**

Within the study period, 184 patients (median age 52 [40–62] years, 122 [63.1%] female) met inclusion criteria with normal motility, complete ESQ-30 questionnaires and no evidence of GERD. Median ESQ-D, ESQ-R, and ESQ-G scores were 4 [0–17], 23 [7–41.75], and 16 [4-36], respectively. Females reported higher symptom scores than males: ESQ-D (7.5 vs 1.5, *p* = .004), ESQ-R (28.0 vs 15.5, *p* = .012), and ESQ-G (19.5 vs 9, *p* = .007). Males demonstrated lower mean DCI (p = .024 and 1918.3 vs 2442.2 mmHg●cm●s, *p* = .029). Increasing age correlated negatively with reflux symptoms (ESQ-R ρ = .178, *p* = .016) and mean DCI (ρ = .156, *p* = .035). No other significant associations between age, manometric parameters, or symptoms were observed.

**Conclusions:**

Symptom profiles in reflux-negative patients with normal esophageal motility vary by age and sex. Women reported greater symptom burden across dysphagia, reflux, and globus domains, while older patients reported fewer reflux symptoms. These findings suggest demographic factors influence symptom perception independent of motility or reflux. Subgroup analyses (e.g., functional heartburn vs functional dysphagia) may clarify pathophysiologic differences and guide individualized management strategies.

**Funding Agencies:**

None

